# Endoscopic Treatment of Anastomotic Biliary Stenosis in Patients With Orthotopic Liver Transplantation

**DOI:** 10.1155/DTE.2.93

**Published:** 1995

**Authors:** Alfredo Rossi, Claudio Grosso, Giulio Zanasi, Pietro Gambitta, Marta Bini, Luciano de Carlis, Raffaele Arcidiacono

**Affiliations:** 1 Gastroenterology and Digestive Endoscopy Unit Niguarda Ca'Granda Hospital Milan Italy; 2 Liver Transplantation Unit Niguarda Ca'Granda Hospital Milan Italy; 3 Via NOTA, 41 Milano 20126 Italy

## Abstract

The choledocho-choledochostomy stricture is one of the most frequent complications occurring after liver transplantation. Today endoscopic retrograde cholangiopancreatography may be considered one of the most common methodologic approaches for the diagnosis; at the same time it provides an effective treatment of the stenosis, avoiding more invasive surgery. Biliary flow through a strictured anastomosis definitely improves after endoscopic stenting which, in most cases, resolves the biliary obstruction syndrome; moreover, the stent could allow restoration of the anatomical and functional integrity of the common bile duct. We have successfully treated eight liver transplanted patients with biliary anastomotic stenosis by endoscopic stenting of the common bile duct or by balloon dilation (one patient). The stents were replaced every 3 to 4 months and then removed after 1 year of follow-up. We observed one patient with acute cholangitis due to the clogging of the prosthetic device.


*Diagnostic and Therapeutic Endoscopy,* 1995, Vol. 2, pp. 93–97
Reprints available directly from the publisher
Photocopying permitted by license only

© 1995 Harwood Academic Publishers GmbH
Printed in Singapore

### Endoscopic Treatment of Anastomotic Biliary Stenosis in Patients with Orthotopic Liver Transplantation

ALFREDO ROSSI, CLAUDIO GROSSO, GIULIO ZANASI, PIETRO GAMBITTA, MARTA BINI, LUCIANO DE CARLIS and RAFFAELE ARCIDIACONO

*Gastroenterology and Digestive Endoscopy Unit, (A.R., C.G., G.Z., P.G., M.B., R.A.) and Liver Transplantation Unit (L.D.C.), Niguarda Ca'Granda Hospital, Milan, Italy*

*(Received April 26, 1995; in final form, May 18, 1995)*

The choledocho-choledochostomy stricture is one of the most frequent complications occurring after liver transplantation. Today endoscopic retrograde cholangiopancreatography may be considered one of the most common methodologic approaches for the diagnosis; at the same time it provides an effective treatment of the stenosis, avoiding more invasive surgery. Biliary flow through a strictured anastomosis definitely improves after endoscopic stenting which, in most cases, resolves the biliary obstruction syndrome; moreover, the stent could allow restoration of the anatomical and functional integrity of the common bile duct. We have successfully treated eight liver transplanted patients with biliary anastomotic stenosis by endoscopic stenting of the common bile duct or by balloon dilation (one patient). The stents were replaced every 3 to 4 months and then removed after 1 year of follow-up. We observed one patient with acute cholangitis due to the clogging of the prosthetic device.

**KEY WORDS:** Biliary endoscopy, common bile duct, liver transplantation, stenting, stricture

### INTRODUCTION

Reconstruction of the common bile duct during a liver transplantation (OLTX) is a very critical stage of the operation: usually it is accomplished by end-to-end choledocho-choledochostomy (CCS) (1). The CCS itself may develop a series of early or late complications, such as bile leaks (subhepatic biloma), sludge or stones, mucocele of the cystic duct remnant, and anastomotic strictures (1–9). In the literature, the overall complication rate ranges from 10 to 25% (1–5,7,9,10); the onset is higher within the first 3 months after OLTX (2–9). Anastomotic stenoses represent one-third of the complications.

A clinically significant stenosis results in a corresponding biliary obstruction syndrome (BOS), which can be detected by specific laboratory findings. In some OLTX patients morphological evidence for a dilated biliary tree above the stenosis may be lacking. In addition, dilation of the recipient common bile duct may be found (1).

Address for correspondence: Alfredo Rossi, M.D., Via NOTA, 41, 20126–Milano, Italy.

Endoscopic retrograde cholangiopancreatography (ERCP) is a commonly used method for morphological and functional evaluation of the biliary ducts after liver transplantation (6,8). In addition, it could provide less invasive treatment of stenosis characterized by impairment of the bile flow.

The aim of our study was to verify the possible therapeutic role of biliary operative endoscopy in OLTX patients with CCS stenosis.

### METHODS AND PATIENTS

Ten of 160 patients with OLTX (6.25%) underwent ERCP because of BOS (Figs. 1 to 4) with or without evident jaundice. Etiological and biological features of patients are shown in Table 1. Serum bilirubin levels ranged from 2.10 to 13.50 mg/dl (mean value = 4.61 mg/dl). Stenosis of the end-to-end CCS was found in eight patients. Only two of them had mildly dilated biliary trees upstream. The following treatments were performed on the eight stenotic patients:

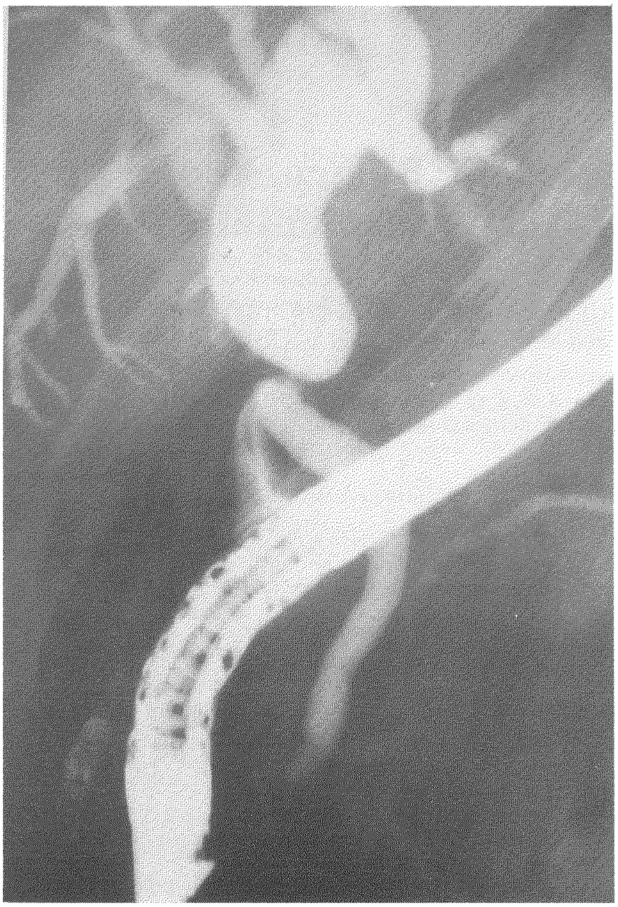

**Figure 1** Stricture of choledocho-choledochostomy in a liver-transplanted patient: dilated biliary tree above the stenosis.

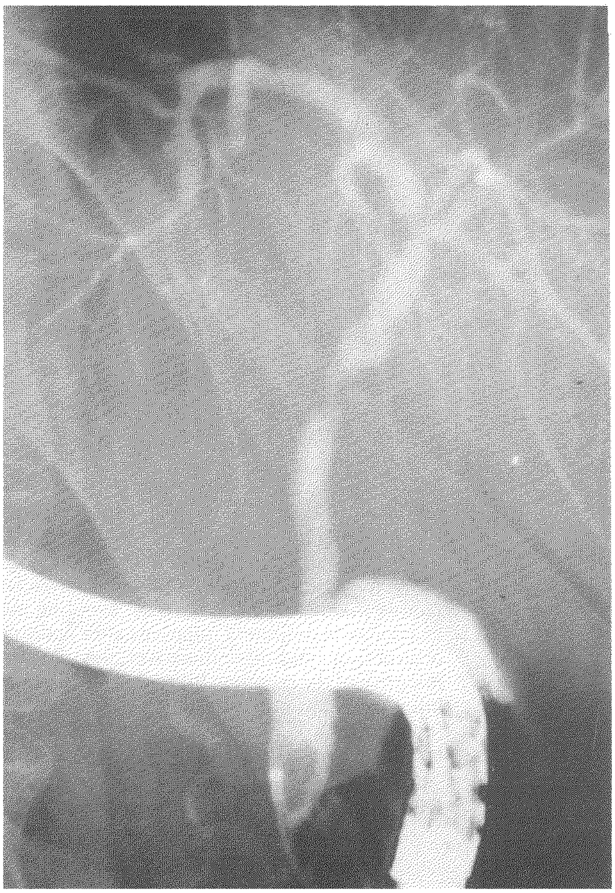

**Figure 2** A strictured choledocho-choledochostomy without dilatation of the upstream biliary ducts. A stone may be seen in the recipient common bile duct.

*One patient*: balloon dilation (PEG-7-38-6, Wilson-Cook Medical Inc., Winston-Salem, NC); the balloon, 8-mm size and filled with distilled water, was inflated at 9 atm pressure for 1 minute.

*Six patients*: stenting with single 10F prosthesis (Cotton-Huibregtse type, Wilson-Cook Medical Inc.) (Fig. 5);

*One patient*: stenting with a double prosthetic device, 10 + 7 F (Cotton-Huibregtse type, Wilson-Cook Medical Inc.).

The balloon dilation and the double stent placement required previous endoscopic papillotomy. Endoscopic procedures were performed with the JF-1 T20 side-viewing duodenoscope (Olympus Corp., Lake Success, NY). Informed consent was obtained.

The efficacy of the biliary drainage was evaluated by improvement of the BOS 7 days after the endoscopic procedure. The follow-up schedule required substitution of the stents every 3 to 4 months and definitive removal after 1 year, unless complications occurred (Fig. 6).

### RESULTS

In all cases the BOS was improved within the 7th day after stenting: jaundice disappeared in seven patients, while in one patient the bilirubin level decreased from 11.4 to 2.3 mg/dl. Table 2 summarizes what happened for each patient during the follow-up period.

Canalization of the stenotic tract was achieved for the patient treated with balloon dilation at the first attempt, and there were no recurrences (control by ERCP at 4.5 months) or complications until the end of his follow-up (9 months). Three of the six patients treated with a single 10F stent finished the established program. These patients

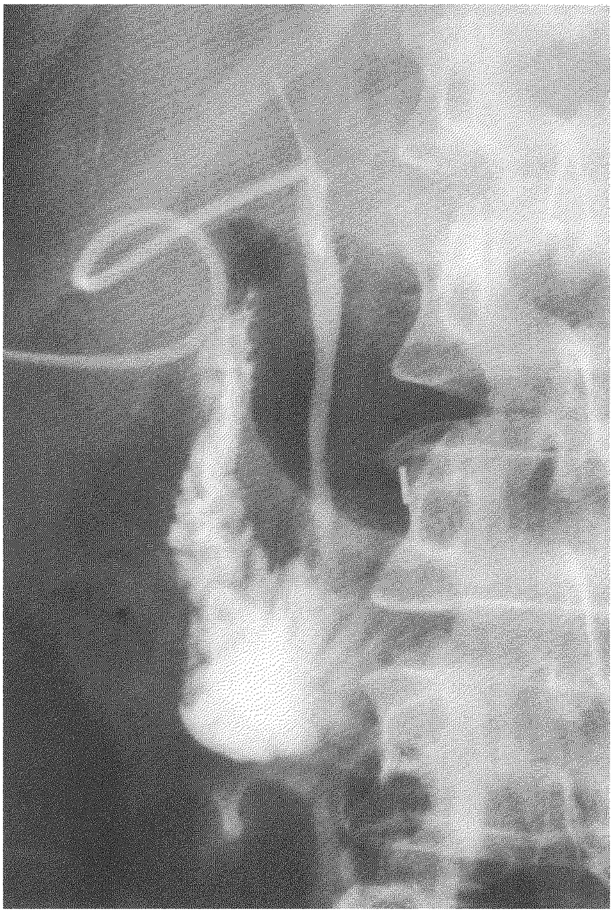

**Figure 3** Severe anastomotic stricture in OLTX patient: trans-Kehr cholangiography shows complete stop of contrast medium.

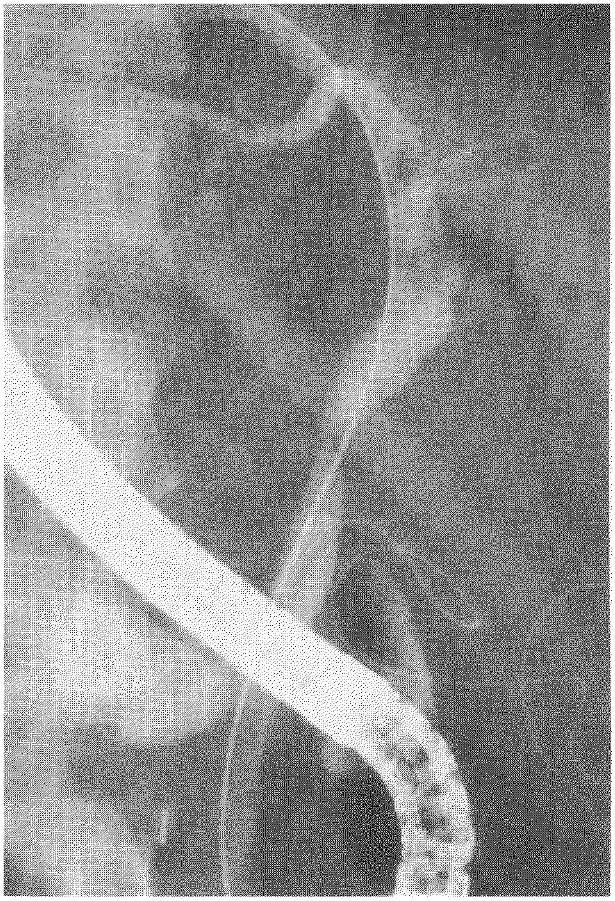

**Figure 4** After ERCP a guide-wire is inserted through the stricture, reaching the left hepatic duct (same patient as Fig. 3).

were always symptom-free, without clinical or biohumoral evidence of BOS. One patient had acute cholangitis 20 days after stenting; clogging, with obstruction of the stent, was found by ERCP. The obstructed device was replaced with a new one of the same size, after which the septic condition rapidly improved. The patient had acute hepatic rejection in the 3rd month of follow-up and was given another transplant. The last two patients have not yet been followed-up for more than 3 months.

The patient treated with double stenting had a replacement with a single 10F stent at 4 months, then another replacement at 7 months. Finally, the stent was removed at 10 months, without any complications and the patient is now symptom-free.

**Table 1** Clinical and Biological Features of 10 OLTX Patients with BOS

| | Value |
|---|---|
| Number of patients | 10 |
| Sex: M/F | 6/4 |
| Age (yr) | |
| Range | 31–55 |
| Mean | 46 |
| Etiology of liver disease | |
| Cirrhosis, hepatitis C virus | 3 |
| Cirrhosis, hepatitis B–D virus | 2 |
| Primary biliary cirrhosis | 1 |
| Cirrhosis + hepatocellular carcinoma | 4 |
| Serum bilirubin level (mg/dl) | |
| Range | 2.10–13.50 |
| Mean | 4.61 |

### DISCUSSION

The onset of evident biliary obstruction in a OLTX patient could immediately lead one to think of a possible hepatic rejection. However, in some cases it may more simply be due to a stenosis of the choledocho-choledochostomy.

Postoperative strictures in OLTX patients seem to be independent of either the most common etiopatho-

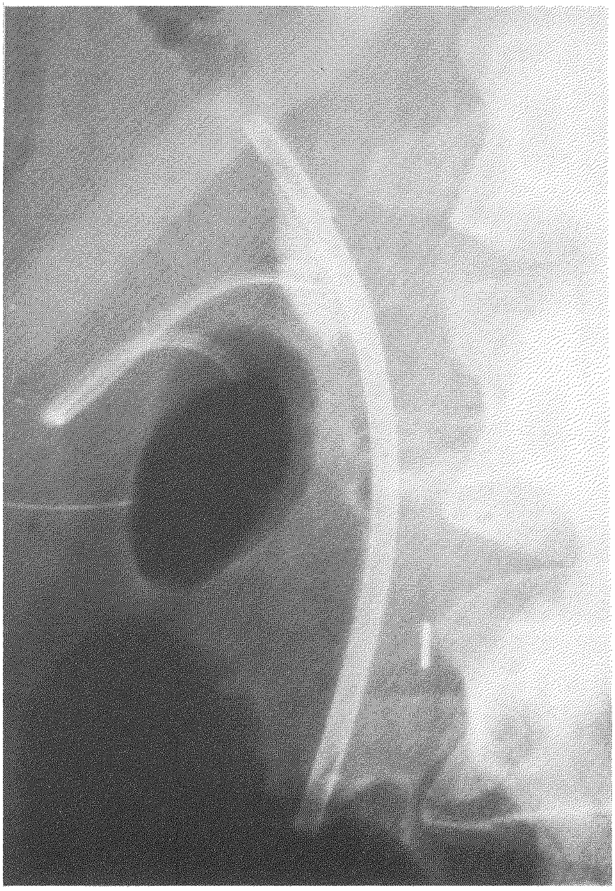

**Figure 5** Stenting is performed by a 10F polyethylene device (same patient as Fig. 3).

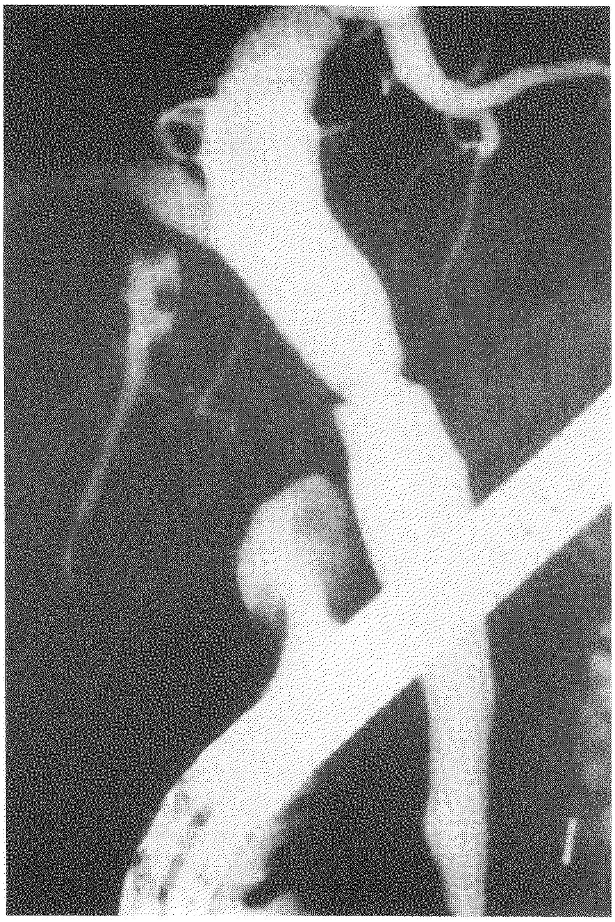

**Figure 6** Twelve months after the first stenting procedure: the last stent is removed and the choledocho-choledochostomy is well canalized (same patient as Fig. 3).

genetic factors connected to the hepatic disease[11] or infectious or pharmacological situations. The main cause of this complication, apart from technical problems, is probably the unfavorable blood supply to the biliary tract.[3] ERCP is therefore an important diagnostic procedure for these patients and moreover it offers an effective and relatively noninvasive treatment of the stenosis itself.[6,12]

The CCS stenosis may be classified as "not neoplastic" and, if so, it could be treated with the same endoscopic procedures that have been codified for the most classical model of "benign stenosis" of the common bile duct, that is, the postcholecystectomy stricture.[13] Following this guideline we used both balloon dilation and, above all, the polyethylene prosthesis. Stenting seems to offer better advantages, as well as a lower risk of complications. This procedure in fact enhances biliary

**Table 2** Endoscopic Treatment and Follow-up of Eight OLTX Patients with Symptomatic Anastomotic Stricture.

| | | | | | |
|---|---|---|---|---|---|
| No. of patients | 1 | | 6 | | 1 |
| Therapy | balloon | | 1 stent | | 2 stent |
| | | 3 | 1 | 2 | |
| Follow-up: | | | | | |
| 1 month | | | Ch * | ⊥ | |
| 2 | | | | | |
| 3 | | * | LRej | | |
| 4 | ERCP | | | | * (1 stent) |
| 5 | | | | | |
| 6 | | | | | |
| 7 | | * | | | |
| 8 | ⊥ | | | | * |
| 9 | | | | | |
| 10 | | * | | | *** |
| 11 | | | | | |
| 12 | | *** | | | |
| 13 | | ⊥ | | | ⊥ |

Abbreviations: Ch, acute cholangitis; LRej, liver rejection; *, stent replacement; ***, stent removal; ⊥, end of follow-up with patient being well.

flow so that the BOS can rapidly improve. Moreover, it may allow the common bile duct to rebuild itself around the stent, which in this way becomes a sort of plastic mold: after 10 to 12 months of stenting, the CCS could be wide enough to permit an adequate biliary flow. In our experience, at the end of follow-up schedule ERCP showed a well-canalized CCS, and patients were clinically asymptomatic. Prolonged clinical observation would be necessary to establish the long-term efficacy of this procedure.

In the majority of our patients, ERCP showed small-sized biliary trees above the stricture. This might be due to early and temporary edema and infiltration of the portal tract of the transplanted liver, although a conclusive explanation is still lacking. However, this finding should be differentiated from the so-called "vanishing bile duct syndrome". This nosological entity, recently described in the literature (14), is defined as disappearance of biliary ducts. It can be seen even histologically and may be considered a particular type of liver rejection. Arterial thrombosis is another pathogenesis to be suspected in the presence of single or multiple stenosis of bile ducts in a transplanted liver.

Because of the above-mentioned small size of the common bile duct, we preferred to use single 10F stents. Only one patient was given a double 10 + 7 F stent. Nevertheless, we did not find any delay or limiting of the canalization of strictures.

Finally, we want to emphasize the need for periodically planned replacement of the stents to reduce the risk of clogging and avoid the consequent cholangitis, which is a particularly dreadful event in OLTX patients treated with immunosuppressive drugs.

### REFERENCES

1. Van Thiel DH, Fagiuoli S, Wright HI, et al. Biliary complications of liver transplantation. Gastrointest Endosc 1993;39:455–460.
2. Bresson-Hadny S, Guerzider P, Miguet JP, et al. Complication in 52 liver transplantations excluding graft rejection. Gastroenterol Clin Biol 1990;14:474–482.
3. Decurtins M, Lachat M, Largiader F. Bile duct reconstruction in liver transplantation. Helv Chir Acta 1990;57819:83–86.
4. Lerut J, Gordon RD, Iwatsuki S, et al. Biliary tract complications in human orthotopic liver transplantation. Transplantation 1987;43:47–51.
5. Letourneau JG, Castaneda-Zuniga WR. The role of radiology in the diagnosis and treatment of biliary complications after liver transplantation. Cardiovas Intervent Radiol 1990;13:278–282.
6. O'Connor HJ, Vickers CR, Buckels JAC, et al. Role of endoscopic retrograde cholangiopancreatography after orthotopic liver transplantation. Gut 1991;32:419–423.
7. Pariente B, Bihet MH, Tammam S, et al. Biliary complications after transplantation in children: role of imaging modalities. Pediatr Radiol 1991;21:175–178.
8. Wolfsen HC, Porayko MK, Hughes RH, et al. Role of endoscopic retrograde cholangiopancreatography after orthotopic liver transplantation. Am J Gastroenterol 1992;87:955–960.
9. Donovan J. Nonsurgical management of biliary tract disease after liver transplantation. Gastroenterol Clin North Am 1993;22:317–336.
10. Vicente E, Perkins JD, Sterioff S, et al. Biliary tract complications following orthotopic liver transplantation. Clin Transplant 1987;1:138–142.
11. Sheng R, Zajko AB, Campbell WL, et al. Biliary strictures in hepatic transplants: prevalence and types in patients with primary sclerosing cholangitis vs those with other liver diseases. Am J Roentgenol 1993;161:297–300.
12. Donovan JP, Sorrell MF, Stratta RJ, et al. Endoscopic placement of expandable prosthesis on therapy for biliary strictures following liver transplantation [Abstract]. Hepatology 1991;14:60A.
13. Davids PHP, Rauws EAJ, Coene PPLO, et al. Endoscopic stenting for . post-operative biliary strictures. Gastrointest Endosc 1992;38:12–18.
14. Hubscher SG, Buckels JA, Elias E, et al. Vanishing bile duct syndrome following liver transplantation—is it reversible? Transplantation 1991;51:1004–1010.

